# Natural history of patients with venous thromboembolism and hereditary hemorrhagic telangiectasia. Findings from the RIETE registry

**DOI:** 10.1186/s13023-019-1172-8

**Published:** 2019-08-09

**Authors:** Antoni Riera-Mestre, José María Mora-Luján, Javier Trujillo-Santos, Jorge Del Toro, José Antonio Nieto, José María Pedrajas, Raquel López-Reyes, Silvia Soler, Aitor Ballaz, Pau Cerdà, Manel Monreal, the RIETE Investigators, Manuel Monreal, Paolo Prandoni, Benjamin Brenner, Dominique Farge-Bancel, Raquel Barba, Pierpaolo Di Micco, Laurent Bertoletti, Sebastian Schellong, Inna Tzoran, Abilio Reis, Marijan Bosevski, Henri Bounameaux, Radovan Malý, Peter Verhamme, Joseph A. Caprini, Hanh My Bui, M. D. Adarraga, M. Agud, M. A. Aibar, M. Alcalde-Manero, J. Alfonso, C. Amado, J. I. Arcelus, A. Ballaz, R. Barba, C. Barbagelata, M. Barrón, B. Barrón-Andrés, A. Blanco-Molina, A. M. Camon, I. Cañas, J. Castro, P. Cerdà, J. de Miguel, J. del Toro, P. Demelo, C. Díaz-Pedroche, J. A. Díaz-Peromingo, I. M. Domínguez, J. C. Escribano, C. Falgá, C. Fernández-Capitán, M. C. Fernández-Criado, M. A. Fidalgo, K. Flores, C. Font, L. Font, I. Furest, M. A. García, F. García-Bragado, A. García-Raso, O. Gavín-Blanco, O. Gavín-Sebastián, A. Gil-Díaz, D. Godoy-Díaz, V. Gómez, C. Gómez-Cuervo, J. González-Martínez, E. Grau, L. Guirado, J. Gutiérrez, L. M. Hernández-Blasco, L. Jara-Palomares, M. J. Jaras, D. Jiménez, M. D. Joya, I. Jou, A. Lalueza, R. Lecumberri, J. Lima, P. Llamas, J. L. Lobo, L. López-Jiménez, M. López-Meseguer, P. López-Miguel, J. J. López-Núñez, R. López-Reyes, J. B. López-Sáez, M. A. Lorente, M. Loring, M. Lumbierres, O. Madridano, A. Maestre, P. J. Marchena, F. Martín-Martos, C. Martínez-Baquerizo, M. A. Martínez-García, M. Mellado, J. Moisés, M. Monreal, M. V. Morales, A. Muñoz-Blanco, J. A. Nieto, M. J. Núñez, M. C. Olivares, P. E. Olivera, C. Ortega, J. Osorio, S. Otalora, R. Otero, M. Panadero-Macia, V. Parra, J. M. Pedrajas, G. Pellejero, C. Pérez-Ductor, G. Pérez-Rus, M. L. Peris, D. Pesantez, J. A. Porras, A. Riera-Mestre, A. Rivas, A. Rodríguez-Cobo, C. Rodríguez-Matute, V. Rosa, C. M. Rubio, P. Ruiz-Artacho, P. Ruiz-Sada, J. C. Sahuquillo, M. C. Sala-Sainz, G. Salgueiro, A. Sampériz, R. Sánchez-Martínez, J. F. Sánchez-Muñoz-Torrero, E. Seguí, S. Soler, S. Suárez, J. M. Suriñach, C. Tolosa, M. I. Torres, J. Trujillo-Santos, F. Uresandi, B. Valero, R. Valle, G. Vidal, C. Vilar, P. Villares, P. Gutiérrez, F. J. Vázquez, A. Vilaseca, T. Vanassche, C. Vandenbriele, P. Verhamme, J. Hirmerova, R. Malý, E. Salgado, I. Benzidia, L. Bertoletti, A. Bura-Riviere, P. Debourdeau, M. C. Courtois, D. Farge-Bancel, H. Helfer, A. Hij, I. Mahé, F. Moustafa, S. Schellong, A. Braester, B. Brenner, I. Tzoran, F. Bilora, C. Bortoluzzi, M. Ciammaichella, F. Dentali, P. Di Micco, P. Ferrazzi, E. Imbalzano, C. Lodigiani, R. Maida, D. Mastroiacovo, N. Mumoli, F. Pace, R. Pesavento, F. Pomero, P. Prandoni, R. Quintavalla, A. Rocci, L. Rota, C. Siniscalchi, E. Tiraferri, A. Tufano, A. Visonà, N. Vo Hong, B. Zalunardo, R. V. Kalejs, D. Kigitovica, A. Skride, M. Bosevski, M. Zdraveska, H. Bounameaux, L. Mazzolai, J. A. Caprini, A. J. Tafur, H. M. Bui

**Affiliations:** 1 HHT Unit and VTE Unit, Internal Medicine Department, Hospital Universitari de Bellvitge, Bellvitge Biomedical Research Institute (IDIBELL), C/ Feixa Llarga s/n. L’Hospitalet de Llobregat, 08907 Barcelona, Spain; 2 Faculty of Medicine and Health Sciences, Universitat de Barcelona, Barcelona, Spain; 3 Department of Internal Medicine, Hospital General Universitario Santa Lucía, Cartagena, Universidad Católica de Murcia, Murcia, Spain; 4 Department of Internal Medicine, Hospital General Universitario Gregorio Marañón, Madrid, Spain; 5 Department of Internal Medicine, Hospital General Virgen De La Luz, Cuenca, Spain; 6 Department of Internal Medicine, Hospital Clínico San Carlos, Madrid, Spain; 7 Department of Pneumonology, Hospital Universitari i Politècnic La Fe, Valencia, Spain; 8 Department of Internal Medicine, Fundació Hospital d’Olot i Comarcal de la Garrotxa, Gerona, Spain; 9 Department of Pneumonology, Hospital De Galdakao, Galdakao, Vizcaya Spain; 10 Department of Internal Medicine, Hospital Germans Trias i Pujol Badalona, Barcelona, Universidad Autónoma de Barcelona, Barcelona, Spain

**Keywords:** Deep venous thrombosis, Hemorrhagic hereditary telangiectasia, Pulmonary embolism, Rare diseases, Venous thromboembolism

## Abstract

**Background:**

Limited data exist about the clinical presentation, ideal therapy and outcomes of patients with hereditary hemorrhagic telangiectasia (HHT) who develop venous thromboembolism (VTE).

**Methods:**

We used the data in the RIETE Registry to assess the clinical characteristics, therapeutic approaches and clinical outcomes during the course of anticoagulant therapy in patients with HHT according to initial presentation as pulmonary embolism (PE) or deep venous thrombosis (DVT).

**Results:**

Of 51,375 patients with acute VTE enrolled in RIETE from February 2009 to January 2019, 23 (0.04%) had HHT: 14 (61%) initially presented with PE and 9 (39%) with DVT alone. Almost half (47.8%) of the patients with VTE had a risk factor for VTE. Most PE and DVT patients received low-molecular-weight heparin for initial (71 and 100%, respectively) and long-term therapy (54 and 67%, respectively). During anticoagulation for VTE, the rate of bleeding events (major 2, non-major 6) far outweighed the rate of VTE recurrences (recurrent DVT 1): 50.1 bleeds per 100 patient-years (95%CI: 21.6-98.7) vs. 6.26 recurrences (95%CI: 0.31–30.9; *p* = 0.020). One major and three non-major bleeding were epistaxis. No patient died of bleeding. One patient died shortly after being diagnosed with acute PE.

**Conclusions:**

During anticoagulation for VTE in HHT patients, there were more bleeding events than VTE recurrences. Most bleeding episodes were non-major epistaxis.

## Introduction

Hereditary hemorrhagic telangiectasia (HHT) or Rendu-Osler-Weber disease is a rare vascular genetic disorder with an estimated prevalence of one in 6000 individuals [1, 2]. Patients with HHT have cutaneous telangiectasia, mostly in the fleshy part of the hands, or in the oral, nasal or gastrointestinal (GI) mucosa, and large arteriovenous malformations (AVMs), mostly in the lungs or liver [3, 4]. These patients may suffer from many complications including bleeding, anemia, iron deficiency, stroke and high-output heart failure [4–6].

The diagnosis of HHT is established on clinical criteria, according to the Curaçao criteria: recurrent epistaxis, cutaneous/mucosal telangiectasia, visceral involvement, and a first line family member with HHT [7]. The diagnosis may be further confirmed by the identification of causative mutations mostly in either the *ENG* (endoglin) or the *ACVRL1* (activin A receptor type II-like 1) genes coding for endoglin and activin receptor-like kinase-1, respectively [2]. These proteins are involved in the vascular signaling pathway of the transforming growth factor (TGF)-beta family [2, 8]. Recurrent epistaxis is the most common symptom, appearing in around 95% of HHT patients by 50 years of age [3, 4, 9]. Although recurrent bleeding events are the hallmark, some patients with HHT may also develop venous thromboembolism (VTE) [9–11]. HHT patients with acute venous thromboembolism (VTE) are perceived to be at high risk for major bleeding if anticoagulant therapy is prescribed, but the evidence on this issue is scarce and based on online interviews [12, 13].

The RIETE (Registro Informatizado Enfermedad TromboEmbólica) registry is an ongoing, multicenter, observational registry of consecutive patients with objectively confirmed acute VTE (ClinicalTrials.gov identifier: NCT02832245). Data from this registry have been used to evaluate outcomes after acute VTE, such as the frequency of recurrent VTE, major bleeding or mortality, and risk factors for these outcomes [14–16]. The rationale and methodology of RIETE have been published elsewhere [17]. In the current study, we aimed to assess the clinical characteristics, therapeutic approaches and clinical outcomes of patients with HHT and VTE, according to initial presentation as pulmonary embolism (PE) or deep venous thrombosis (DVT).

## Methods

### Inclusion criteria

Consecutive patients with acute, symptomatic DVT or PE confirmed by objective tests (compression ultrasonography or contrast venography for DVT; helical CT-scan of the chest, ventilation-perfusion lung scintigraphy or angiography for PE) were enrolled in RIETE. Patients were excluded if they were currently participating in a blind therapeutic clinical trial. All patients (or their relatives) provided written or oral consent for participation in the registry, in accordance with local ethics committee requirements.

### Study design

For this study, only patients with HHT were considered. This information was added to the RIETE data abstraction forms in February 2009. Therefore, only patients recruited after this date were eligible for the current study. Collected data were appropriately made anonymous and each patient was identified by a unique alphanumeric identification code. HHT patients were divided according to VTE index event as PE or DVT. Primary outcome was the risk for VTE recurrences and major or non-major bleeding occurring during the course of anticoagulation. Secondary outcomes were fatal PE and fatal bleeding. Bleeding events were classified as ‘major’ if they were overt and required transfusion of two or more red blood cell units, or were retroperitoneal, spinal or intracranial. All clinically evident bleeding not considered major, were classified as ‘non-major’. Fatal PE, in the absence of autopsy, was defined as any death appearing < 10 days after PE diagnosis, in the absence of any alternative cause of death. Fatal bleeding was defined as any death occurring < 10 days after a major bleeding episode, in the absence of any alternative cause of death. For preparation of this manuscript, we followed the Strengthening the Reporting of Observational Studies in Epidemiology (STROBE) statement guidelines for observational cohort studies [18].

### Study variables

The following parameters were recorded in RIETE: patient’s baseline characteristics; clinical status including any coexisting or underlying conditions, recent major bleeding, anemia or renal insufficiency; risk factors for VTE; the treatment received upon VTE diagnosis; concomitant drugs and the outcomes during the course of therapy. For normotensive patients with PE, stratification using the simplified version of the Pulmonary Embolism Severity Index (sPESI) was assessed [15]. Immobilized patients were defined as non-surgical patients who had been immobilized (i.e., total bed rest with or without bathroom privileges) for ≥4 days in the 2-month period prior to VTE diagnosis. Surgical patients were defined as those who had undergone an operation in the 2 months prior to VTE. Active cancer was defined as newly diagnosed cancer (< 3 months before) or when receiving anti-neoplastic treatment of any type (i.e., surgery, chemotherapy, radiotherapy or hormonal therapies). Recent bleeding was considered in those patients suffering major bleeding < 30 days prior to VTE. Anemia was defined as hemoglobin levels < 13 g/dL for men and < 12 g/dL for women.

### HHT additional information

When preparing this study, we attempted to gather as much specific information on HHT as possible, through personal communication with the attending doctors. The study coordinating center contacted with the investigators and tried to retrospectively calculate the Epistaxis Severity Score (ESS) at the index VTE event. ESS is an on-line tool that estimates the severity of epistaxis according to clinical data occurring during last 3 months. Epistaxis is considered moderate or severe if ESS results > 4 or > 7 points, respectively [19]. We also attempted to assess the genetic study for HHT, specifically for mutations in *ENG* or *ACVRL1* genes, and information about the Curaçao criteria [2, 7, 8].

### Treatment and follow-up

Patients were managed according to the clinical practice of each participating hospital (i.e., there was no standardization of treatment). The type, dose and duration of anticoagulant therapy were recorded. After VTE diagnosis, all patients were followed-up in the outpatient clinic for at least 3 months. During each visit, any signs or symptoms suggesting VTE recurrences or bleeding complications were noted. Each episode of clinically suspected recurrent VTE was investigated by using diagnostic tests as appropriate. Most outcomes were classified as reported by the clinical centers. However, if staff at the coordinating center were uncertain how to classify a reported outcome, that event was reviewed by a central adjudicating committee (less than 10% of events).

### Statistical analysis

Categorical variables were compared between PE and DVT patients with HHT using the chi-square test (two-sided) and Fisher’s Exact Test (two-sided). Odds ratio (and its 95% confidence interval) was calculated for categorical variables. Continuous variables were compared using Student t test. The incidence rate of adverse events was calculated as events per 100 patient-years of treatment and compared, according to the initial presentation of the VTE episode (PE vs. DVT), as rate ratios, both with a 95% confidence interval. A *P*-value of < 0.05 was chosen as the cut off for statistical significance. Statistical analyses were conducted using SPSS for Windows Release (version 20, SPSS Inc. Chicago, Illinois).

## Results

Of 51,375 patients enrolled in RIETE from February 2009 to January 2019, 23 (0.04%) had HHT. Of these, 13 (56.5%) were male, mean age was 61 ± 17 years, 14 (61%) presented initially with PE and 9 (39%) presented with DVT alone. Eighteen patients (78%) had a history of recurrent epistaxis, and three (13%) had suffered a major bleeding (all epistaxis) in the last 30 days before the index VTE event. There was available information to calculate the ESS in 16 (69%) patients, and mean ESS was 4.4 ± 2.4 (0. 9-8.4) points. Genetic study was positive for ENG in 5 (22%) patients, for ACVRL1 in 2 (9%) and one (4.5%) patient had negative results (but had 3 of 4 Curaçao criteria).

Six patients (26%) developed the VTE after being immobilized for ≥4 days (because of decompensated chronic lung disease 2, trauma 2, ischemic stroke 1, pneumonia 1), two (9%) after undergoing surgery (hernia disc repair 1, hip fracture 1), one patient (4.5%) had cancer and two (9%) had a history of prior VTE. Only three patients had received prophylaxis before the index VTE: two patients admitted in the hospital because of decompensated chronic lung disease and one patient with hip fracture. Twelve (52%) patients had anemia at baseline, six (26%) had creatinine clearance levels < 60 mL/min, three (13%) had angiodysplasia in the GI tract and one (4.5%) patient had a GI ulcer. All patients had normal prothrombin test and activated partial thromboplastin time. There were no significant differences in the clinical characteristics between patients who initially presented with PE or DVT. All 14 patients initially presenting with PE were admitted in hospital and 7 (50%) were considered to be at high risk according to sPESI score (Table 1).

**Table 1:** Clinical characteristics of 23 patients with HHT and acute VTE

	Pulmonary embolism	Deep vein thrombosis	Odds ratio (95% CI) / *P* value
*Patients, N*	14	9	
Clinical characteristics,			
Male gender	6 (43%)	7 (78%)	0.21 (0.03–1.43)
Mean age (years±SD)	60 ± 16	62 ± 19	*P* = 0.708
Mean body weight (kg ± SD)	87 ± 28	72 ± 9.7	*P* = 0.145
Data on HHT,^a^			
Spontaneous, recurrent epistaxis (*n* = 18)	12 (86%)	6 (67%)	
Epistaxis severity score (*n* = 9 and 7)	3.5 (0. 9-8.4; ±2.1)	5.4 (2. 7-8.1;±2.3)	*P* = 0.097
ESS > 4	3 (21%)	4 (44%)	*P* = 0.615
ESS > 7	1 (7%)	3 (33%)	*P* = 0.262
Genetic study (*n* = 11)			
ENG mutations	2 (14%)	3 (33%)	
ACVRL1 mutations	1 (7%)	1 (11%)	
Negative	1 (7%)	0	
Family history (*n* = 16)	10 (71%)	6 (67%)	
Risk factors for VTE,			
Recent surgery	1 (7%)	1 (11%)	0.62 (0.03–11.28)
Recent immobilization ≥4 days	4 (29%)	2 (22%)	1.40 (0. 20-9.87)
Cancer	1 (7%)	0	–
Prior VTE	1 (7%)	1 (11%)	0.62 (0.03–11.28)
None of the above	6 (43%)	6 (67%)	0.38 (0.07–2. 15)
Underlying conditions,			
Prior ischemic stroke	3 (21%)	0	–
Atrial fibrillation	3 (21%)	0	–
Gastroduodenal ulcer	1 (7%)	0	–
Angiodisplasia in the GI tract	3 (21%)	0	–
Recent (< 30 days) major bleeding	0	3 (33%)	–
Laboratory data,			
Anemia	6 (43%)	5 (56%)	0.60 (0. 11-3.25)
CrCl levels < 30 mL/min	0	0	–
CrCl levels 30–60 mL/min	2 (14%)	3 (33%)	0.33 (0.04–2.56)
sPESI,			
≥ 1 points	7 (50%)	–	–

*Abbreviations: SD* standard deviation, *HHT* hemorrhagic hereditary telangiectasia, *ENG* endoglin gene, *ACVRL1* activin A receptor type II-like 1 gene, *VTE* venous thromboembolism, *GI* gastrointestinal, *sPESI* simplified version of the Pulmonary Embolism Severity Index, *CrCl* creatinine clearance levels; 95%CI, 95% confidence intervals

^a^ Retrospective information added, not included in the RIETE Registry

Median duration of anticoagulant therapy was 203 days in patients with PE and 164 days in those with DVT. Most (71%) PE and all (100%) DVT patients were initially treated with low-molecular-weight heparin (LMWH), but four (29%) and two (22%) patients respectively received less than 100 IU/kg/day. Among the remaining patients with PE, two (14%) were prescribed unfractionated heparin, one (7%) rivaroxaban and one (7%) patient received an inferior vena cava filter without anticoagulant therapy. For long-term therapy, 7 (54%) PE patients and 6 (67%) DVT patients continued on long-term LMWH therapy, but one (8%) and two (22%) of these patients received less than 100 IU/kg/day, respectively. Among the remaining patients, five (38%) and three (33%) switched to vitamin K antagonists (VKAs), respectively, and one (8%) PE patient continued on rivaroxaban (Table 2).

**Table 2:** Treatment strategies

	Pulmonary embolism	Deep vein thrombosis	Odds ratio (95% CI) / *P* value
*Patients, N*	14	9	
*Duration of therapy,*			
Mean days (SD)	260 ± 254	243 ± 195	*P* = 0.867
Median days (IQR)	171 (111–335)	177 (86–475)	*P* = 0.999
*Initial therapy,*			
Unfractionated heparin	2 (14%)	0	–
Low-molecular-weight heparin	10 (71%)	9 (100%)	–
Mean LMWH dose (IU/kg/day)	132 ± 50	151 ± 59	*P* = 0.474
LMWH doses < 100 IU/kg/day	4 (29%)	2 (22%)	2.33 (0.31–17.55)
Rivaroxaban	1 (7%)	0	–
Inferior vena cava filter (without anticoagulant therapy)	1 (7%)	0	–
*Long-term therapy,*	13	9	
Low-molecular-weight heparin	7 (54%)	6 (67%)	0.58 (0. 10-3.40)
Mean LMWH dose (IU/kg/day)	135 ± 35	119 ± 43	*P* = 0.491
LMWH < 100 IU/kg/day	1 (8%)	2 (22%)	0.27 (0.02–3.52)
Vitamin K antagonists	5 (38%)	3 (33%)	1.25 (0. 21-7.41)
Rivaroxaban	1 (8%)	0	–
No anticoagulant drugs	0	0	–

*Abbreviations: SD* standard deviation, *IQR* interquartile range, *LMWH* low-molecular-weight heparin, *IU* international units, 95%CI, 95% confidence intervals

During the course of anticoagulation, (mean, 260 ± 254 days for PE and 243 ± 195 days for DVT), the rate of bleeding events (major bleeding 2 episodes, non-major 6) far outweighed the rate of VTE recurrences (recurrent DVT one episode): 50.1 bleeds per 100 patient-years (95%CI: 21.6-98.7) vs. 6.26 recurrences (95%CI: 0.31–30.9; *p* = 0.020). One patient had a major bleeding in the GI tract 534 days after PE diagnosis while receiving VKAs, and one patient had a major epistaxis 32 days after the diagnosis of DVT while receiving LMWH at < 100 IU/kg/day. Three non-major bleeds were epistaxis. All four patients with epistaxis had an ESS > 4. No patient died of bleeding. Among DVT patients, one patient presented a DVT recurrence 227 days after the diagnosis, while receiving vitamin K antagonists. One patient died of respiratory insufficiency due to hospital-acquired pneumonia 4 days after being diagnosed with acute PE, while receiving unfractionated heparin. She did not undergo repeat tests to exclude recurrent PE, so we cannot rule out the diagnosis of fatal PE (Table 3).

**Table 3:** Clinical outcomes during the course of anticoagulant therapy

	Pulmonary embolism		Deep vein thrombosis		Rate ratio (95%CI)
	N	Events per 100 patient-years	N	Events per 100 patient-years	
*Patients, N*	14		9		
Recurrent PE	0	–	0	–	–
Recurrent DVT	0	–	1	16.67 (0. 22-92.72)	–
Major bleeding	1	10.02 (0. 13-55.75)	1	16.67 (0. 22-92.72)	0.60 (0.04–9.61)
*Site of major bleeding (days)* ^*a*^ *,*					
Epistaxis (32^b^)	0	–	1	16.67 (0. 22-92.72)	–
GI (534)	1	10.02 (0. 13-55.75)	0	–	–
Non-major bleeding	3	30.06 (6.04–87.83)	3	50 (10.05–146.1)	0.60 (0. 12-2.98)
*Site of minor bleeding (days)* ^*a*^ *,*					
Epistaxis (9,31,179)	3	30.06 (6.04–87.83)	0	–	–
Others (0^b^,5,50)	0	–	3	50 (10.05–146.1)	–
Death	1	10.02 (0. 13-55.75)	0	–	–
*Causes of death,*					
Respiratory insufficiency	1	10.02 (0. 13-55.75)	0	–	–

*Abbreviations: PE* pulmonary embolism, *DVT* deep vein thrombosis; 95%CI, 95% confidence intervals, *GI* gastrointestinal

^a^ Days from index VTE diagnosis to bleeding event

^b^ Receiving LMWH < 100 IU/kg/day

## Discussion

Since HHT is a rare disorder, the natural history of VTE in these patients, and the optimal therapy are not well known. The only two studies that analyze the effect of anticoagulation in HHT patients were not based on objectively confirmed diagnosis and outcomes of VTE but on on-line surveys [12, 13]. The first one was an on-line questionnaire about the use of antiplatelet or anticoagulant agents in HHT patients [12]. A worsening of their usual epistaxis was referred in 86 (57.3%) of 150 patients with HHT who received anticoagulant therapy for different reasons [12]. In the second study, 20% of 20 patients with self-reported diagnosis of VTE who received anticoagulant therapy had to withdraw anticoagulation because of bleeding [13].

Our findings, obtained from the largest series of HHT patients with objectively confirmed VTE, reveal that one in every 3 (35%) patients bled during the course of anticoagulant therapy, though no bleed was fatal. This may be due to the fact that most bleeds came from nasal or GI tract telangiectasia and not from large vascular malformations (i.e. in the liver, lung or brain), that are common in HHT patients. The ESS seems to detect those patients at an increased risk to develop epistaxis, as all four patients with epistaxis during anticoagulation had a ESS > 4. ENT management to optimize measures to reduce epistaxis could allow to better tolerate anticoagulation [3, 20]. Other options, such as tranexamic acid, topical or systemic estrogens, thalidomide or bevacizumab, should be weighted against the risk of VTE for these drugs [3, 21–24].

Eighteen patients in our cohort (78%) had prior epistaxis, five had renal insufficiency, three had documented angiodisplasia in the GI tract and one patient had a gastroduodenal ulcer. To what extent the use of anticoagulant therapy could have influenced on the risk for bleeding is unknown. Two of the three patients with major epistaxis in the 30 days before the index VTE repeated epistaxis during the course of anticoagulation: one of these had a major re-bleeding. Then, we cannot assume that repeated epistaxis that happened during the course of anticoagulant therapy were exclusively related to anticoagulation, as epistaxis is an inherent feature of HHT [3, 4, 9]. However, anticoagulation can increase the duration and severity of epistaxis [12]. Unfortunately, we have no monitoring ESS during anticoagulant treatment to analyze this association.

In spite of the bleeding risk, some HHT patients also suffer from thrombotic complications [10, 25–27]. Six patients in our cohort developed the index VTE after being immobilized for an acute medical illness and two had recent surgery, but only three of them did receive VTE prophylaxis. We hypothesize that the attending doctors might have been concerned about the risk for bleeding. In the on-line survey of HHT patients with self-reported VTE, 76% of them had a risk factor for VTE [13]. There is consistent evidence on the reduction of VTE using prophylaxis in at-risk patients, with very low increase in the risk for bleeding [28], but we do not know the potential benefit of VTE prophylaxis in at-risk patients with HHT. Further research is necessary to define prophylactic strategies in this frequently hospitalized HHT population. For these provoked VTE events, anticoagulant therapy for only 3 months is recommended [29].

Our study has a number of limitations. First, the proportion of patients with HHT in our cohort was most likely conservative because some cases might have been missed. Second, we were not able to retrospectively collect genetic study and reassess Curaçao criteria in all patients [7, 8]. Nonetheless, the high percentage of recurrent epistaxis and first degree relatives with HHT in our patients, support the HHT diagnosis. Third, our study was not designed for assessment of comparative effectiveness of management strategies in patients with HHT. Since all patients with HHT in our cohort received anticoagulant therapy, we are unable to compare the potential advantages of the different therapeutic approaches. However, our study provides important insights into the outcomes of HTT patients with VTE. Fourth, the ESS has been retrospectively calculated after some time, being a likely cause of ascertainment bias. Finally, although many of the studies from the RIETE registry were designed with broad or detailed a priori plans, HHT is a rare disease and the decisions to explore several of the data elements were based on post-hoc plans and analyses. However, to our knowledge, these data represent the largest series of patients with HHT and objectively confirmed VTE. Moreover, the main strengths of our study are the strict and objectively diagnostic criteria for VTE, the long-term follow-up period and the objectively established outcomes reported (bleeding, recurrent symptomatic VTE and mortality).

## Conclusions

In conclusion, the use of anticoagulant therapy for VTE in patients with HHT may certainly increase the risk for bleeding, but it barely influences on mortality. Most bleeds come from nasal or digestive telangiectasia and not from large HHT-related vascular malformations. So, although bleeding events are more frequent than VTE recurrences, the use of anticoagulation was not associated with fatal bleeding. PE is the most common presentation of VTE in patients with HHT. Almost half of our HHT patients had a risk factor for VTE. Patients with HHT should be carefully evaluated for nosebleeds when needing anticoagulant treatment. The optimal type, dose and duration of anticoagulant therapy for VTE in patients with HHT needs further investigation.

## Data Availability

The datasets used and/or analysed during the current study are available from the corresponding author on reasonable request.

## Abbreviations

95%CI: 95% confidence interval
*ACVRL1*: Activin A receptor type II-like 1 gene
CrCl: Creatinine clearance
DVT: Deep venous thrombosis
*ENG*: Endoglin gene
ESS: Epistaxis Severity Score
GI: Gastrointestinal
HHT: Hereditary hemorrhagic telangiectasia
IQR: Interquartile range
IU: international units
LMWH: Low-molecular-weight heparin
OR: Odds ratio
PE: Pulmonary embolism
RIETE: Registro Informatizado Enfermedad TromboEmbólica
SD: Standard deviation
sPESI: simplified Pulmonary Embolism Severity Index
TGF: Transforming growth factor
VKAs: Vitamin K antagonists
VTE: Venous thromboembolism
